# Estimated impact of COVID-19 on preventive care service delivery: an observational cohort study

**DOI:** 10.1186/s12913-021-07131-7

**Published:** 2021-10-16

**Authors:** Scott Laing, Sharon Johnston

**Affiliations:** 1 University of Ottawa Department of Family Medicine, Telfer School of Management, Ottawa, Canada; 2 University of Ottawa Department of Family Medicine, Institut du Savoir Montfort, Bruyère Research Institute, Ottawa, Canada

## Abstract

**Background:**

COVID-19 has caused significant healthcare service disruptions. Surgical backlogs have been estimated but not for other healthcare services. This study aims to estimate the backlog of preventive care services caused by COVID-19.

**Methods:**

This observational study assessed preventive care screening rates at three primary care clinics in Ottawa, Ontario from March to November 2020 using data from 22,685 electronic medical records. The change in cervical cancer, colorectal cancer, and type 2 diabetes screening rates were crudely estimated using 2016 census data, estimating the volume of key services delayed by COVID-19 across Ontario and Canada.

**Results:**

The mean percentage of patients appropriately screened for cervical cancer decreased by 7.5% (− 0.3% to − 14.7%; 95% CI), colorectal cancer decreased by 8.1% (− 0.3% to − 15.8%; 95% CI), and type 2 diabetes decreased by 4.5% (− 0.2% to − 8.7%; 95% CI). Crude estimates imply 288,000 cervical cancer (11,000 to 565,000; 95% CI), 326,000 colorectal cancer (13,000 to 638,000; 95% CI), and 274,000 type 2 diabetes screenings (13,000 to 535,000; 95% CI) may be overdue in Ontario. Nationally the deficits may be tripled these numbers. Re-opening measures have not reversed these trends.

**Interpretation:**

COVID-19 decreased the delivery of preventive care services, which may cause delayed diagnoses, increased mortality, and increased health care costs. Virtual care and reopening measures have not restored the provision of preventive care services. Electronic medical record data could be leveraged to improve screening via panel management. Additional, system-wide primary care and laboratory capacity will be needed to restore pre-COVID-19 screening rates.

**Supplementary Information:**

The online version contains supplementary material available at 10.1186/s12913-021-07131-7.

## Introduction

COVID-19 has strained our healthcare system and workforce in unprecedented ways and these effects are gradually being understood. Ministry of Health directives significantly reduced the volume of surgical procedures completed since March 2020 [1]. However, COVID-19’s impact on health care delivery extends beyond the operating room. Screening volumes decreased drastically shortly after the start of the COVID-19 pandemic and in many cases have not returned to baseline levels [2]. Yet the volume of patients that are now due for screening according to clinical guidelines has yet to be quantified. Screening services are important for early cancer detection and treatment. Delays in cancer diagnosis and treatment significantly increase mortality rates [3]. Much of this screening is performed by primary care providers in community clinics. The Ontario Ministry of Health has advised that community clinics limit in-person appointments, favouring virtual care assessments instead [4]. Many preventive care services do not require in-person assessments and may still be provided through virtual care [5]. However, many provincial screening programs were paused at the beginning of the pandemic [6–9]. In Ontario, restrictions on colorectal cancer (CRC) screening with fecal immunochemical testing (FIT) lasted almost six months, lifting for high priority patients as of August 25, 2020 [10].

While non-official reports indicate that cancer screening has decreased, there is still limited data that quantifies the volume of delayed preventive care services. Electronic medical record (EMR) data could help provide insight into this deficit [11]. EMRs provide a longitudinal history of patients’ health and statuses, including provision of preventive care services [11]. Primary care EMR data uniquely combines internally produced data with imported data from laboratories, imaging centers, specialists, and acute care [12]. Accordingly, primary care EMR data may provide timely information on health outcomes and service delivery for health system planning and research purposes. This study utilized primary care EMR data to quantify the impact of COVID-19 on the delivery of preventive care services, namely cervical cancer, CRC, and type 2 diabetes mellitus (T2DM) screening. It is expected that the number of patients screened has decreased since COVID-19 restrictions began.

## Methods

### Preventive care data sources

This was an observational cohort study. Data was extracted from three urban primary care clinics in Ottawa, Ontario, Canada. Two clinics were academic family health teams, and one was a community family health organization. All three clinics used PS Suites, which is provided by TELUS Health [13]. A scheduled data export was developed using PS Suites’ built-in scheduled reports functionality [12]. The scheduled report identified all active patient records and output each patient’s age, gender, past medical history, and select preventive care data in a tab delimited file. The scheduled reports ran every week from March 15, 2020, until November 29, 2020. Preventive care data necessary to determine compliance with the Canadian Task Force for Preventive Health Care’s (CTFPHC) recommendations were exported. The number of months since the latest Papanicolaou smear was exported for cervical cancer screening and are represented by four data points [14]. The number of months since the latest fecal occult blood test (FOBT), fecal immunochemical test (FIT), sigmoidoscopy, and colonoscopy were exported for CRC screening [15]. The number of months since and results of the latest hemoglobin A1c (HbA1c) and fasting blood sugar (FBS) were exported for T2DM screening [16]. See Additional file 4 for details of exported data. A separate manual review of 900 charts was completed to validate the exported data and confirmed data accuracy. These 900 charts were randomly selected from consenting providers’ patient panels. The primary investigator first reviewed 300 charts manually and identified which screening tests were due according to the low-risk guidelines used here. The manual chart review was then compared to the algorithm used in this study. Inconsistencies were identified from these 300 charts and the algorithm adjusted before an additional 600 charts were reviewed. This validation has not yet been published but is available upon request from the authors.

### Determining the impact on screening rates

Screening statuses for cervical cancer, CRC, and T2DM were determined from the exported EMR data. The analysis was completed using a Python 3.8 script, which analyzed the exported tab delimited files. The Python script determined each patients’ screening eligibility in agreement with the latest screening recommendations for cervical cancer [14], CRC [15], and T2DM [16, 17] (Table 1). Since insufficient data was available to calculate the T2DM risk (CANRISK score) [18] a minimum screening interval of 3 years was used instead, which aligns with both the CTFPHC [16] and Diabetes Canada [17] guidelines. Patients considered high risk or ineligible for screening were excluded from the analysis. Full details of the inclusion and exclusion criteria algorithm are available in Additional files 1, 2, and 3.

**Table 1:** Summarized Canadian Task Force for Preventive Health Care’s screening recommendations

Screening Type	Age (years)	Sex	Interval
Cervical Cancer [14]	25 to 69	Female	3 years
Colorectal Cancer [15]	50 to 74	Female and Male	2 years (FOBT and FIT) 10 years (sigmoidoscopy and colonoscopy)
Type 2 Diabetes^a^	≥ 40	Female and Male	1 to 5 years based on risk [16] 3 years [17]

^a^The screening interval for type 2 diabetes was selected as 3 years for this study as CANRISK scores were not available [18]. This aligns with the Diabetes Canada recommendations for average risk individuals over 40 years old [17]

After inclusion and exclusion criteria were applied, each primary care providers’ patient panel was assessed, determining the percentage of patients that were up to date for cervical cancer, CRC, and T2DM screening. If screening was completed within the recommended interval, then that patient was counted as up to date for that screening maneuver.

$$ Provide{r}^{\prime }s\  percentage\ of\ patients\  up\  to\ date=\frac{\# of\ patients\  up\  to\ date}{\# of\ patients\ eligible}\ast 100 $$

Once the percentage of patients up to date for the three screening tests was determined for each provider’s patient panel, then the mean percentage of patients up to date was determined for all providers. The mean percentage was determined to keep each provider’s screening rates confidential. The weekly mean percentages of patients up to date on preventive care screening were then graphed chronologically, including key dates from Ontario’s COVID-19 response [19]. Finally, the change in mean percentage of patients up to date for screening between March 15, 2020, and November 29, 2020, was determined.

The impact of COVID-19 on in-person preventive care (cervical cancer screening), provincial screening programs (CRC screening), and screening that could be delivered through virtual care without restriction (T2DM screening) was estimated based on the change in mean from the beginning of the pandemic response [5].

### Crude estimates of the provincial and National Preventive Care Service Deficits

Crude estimates of the preventive care service deficits for Ontario and Canada were calculated based on the study clinics’ experience with service disruptions due to COVID-19. Census data from 2016 [20] were used to estimate the number of people in Ontario and Canada that are due for cervical cancer, CRC, and T2DM screening from baseline levels.

### Statistical analysis

The 95% confidence intervals (CI) for the mean percentage of all providers’ patients that are up to date on cervical cancer, CRC, and T2DM screening were determined using a normal approximation method.

### Ethics approval

Patient consent was not required as was determined by the Bruyère Research Ethics Board at Bruyère Hospital in Ottawa, Ontario. REB Ethics Number: #M16–20-045. All required privacy and security measures were followed to maintain patient confidentiality.

## Results

### Practice demographics

The demographics of the three clinics is presented in Tables 2 and 3. Data was extracted from 22,685 active patients on a weekly basis across 29 providers. On November 29, 2020, there were 6754 patients eligible for cervical cancer screening and 492 were excluded since they met high risk or exclusion criteria according to Canadian screening guidelines. A total of 7168 were eligible for colorectal cancer screening and 200 were excluded. Lastly, 10,933 met eligibility criteria for type 2 diabetes screening and 1469 met the exclusion criteria. The numbers from March 15, 2020, were also reported for reference.

**Table 2:** Study clinic, Ontario, and Canada demographics

Age	Clinic Population (n)	Ontario Population (%)	Expected Ontario (n)	Χ^**2**^ Statistic Ontario	Canadian Population (%)	Expected Canada (n)	Χ^**2**^ Statistic Canada
0	1131	4.9	1176	0.29	5.1	1225	0.22
5 to 9	1257	5.2	1275	5.87	5.4	1302	1.12
10 to 14	1245	5.4	1273	0.47	5.5	1241	0.05
15 to 19	999	5.8	1369	74.67	5.5	1308	51.85
20 to 24	1198	7.1	1509	101.44	6.5	1447	54.42
25 to 29	1454	7.3	1475	25.33	7.0	1475	9.9
30 to 34	1424	7.1	1459	20.2	7.0	1503	17.13
35 to 39	1575	6.7	1421	1.38	6.9	1477	0.02
40 to 44	1600	6.3	1471	23.09	6.5	1455	11.31
45 to 49	1525	6.3	1581	5.64	6.3	1523	6.73
50 to 54	1426	6.6	1782	2.83	6.4	1728	0.89
55 to 59	1753	7.3	1669	6.05	7.2	1691	8.07
60 to 64	1594	6.5	1427	8.78	6.7	1478	2.85
65 to 69	1386	5.5	1244	17.69	5.7	1273	6.54
70 to 74	1209	4.6	892	28.53	4.7	917	18.9
75 to 79	795	3.1	663	10.18	3.2	660	6.36
80 to 84	507	2.2	491	0.46	2.1	484	1.09
85 to 89	331	1.4	318	0.92	1.4	312	1.57
90 to 94	198	0.7	149	13.92	0.7	144	16.89
95 to 99	63	0.2	36	7.2	0.2	37	7.2
100 +	15	0.0	5	13.5	0.0	5	9.14
Total	22,685	100.0	22,685	368.44	100.0	22,685	232.25
*P* value				< 0.001			< 0.001

Chi square goodness of fit test was completed and shows there are differences between the clinic population and both Ontario as well as Canada (*P* < 0.001)

**Table 3:** Study Clinic Patient Demographics

		Date	
		Mar 15, 2020	Nov 29, 2020
		Number (n)	Number (n)
Total Number of Patients		22,648	22,685
Total Number of Providers		29	29
Type of Screening	Eligibility		
Cervical Cancer Screening	Low Risk Eligible	6765	6754
	Excluded	495	492
	Total	7260	7246
Colon Cancer Screening	Low Risk Eligible	7170	7168
	Excluded	201	200
	Total	7170	7368
Diabetes Screening	Low Risk Eligible	10,897	10,993
	Excluded	1474	1469
	Total	12,371	12,402

Indicates the total number of patients and providers included in this study. The number of patients eligible for low-risk screening and excluded due to meeting the exclusion criteria (see Appendices for details) were also reported

### Impact of COVID-19 on mean preventive care screening rates

The period of data collection covered 38 weeks from March 15, 2020, until November 29, 2020. Two weeks of data were excluded due to errors in the scheduled data export causing incomplete data to be exported.

**Fig. 1:**
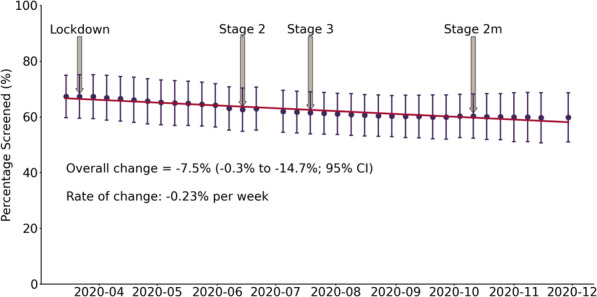
Cervical Cancer Screening Rates. Shows the mean percentage (percentage screened ± SD) of eligible patients up to date for cervical cancer screening each week (*n* = 6765 March 15, 2020; *n* = 6754 November 29, 2020). For reference the lockdown and re-opening stages have been identified. Lockdown = March 20, 2020, Stage 2 = June 12, 2020, Stage 3 = July 17, 2020, and Modified Stage 2 = October 13, 2020

**Fig. 2:**
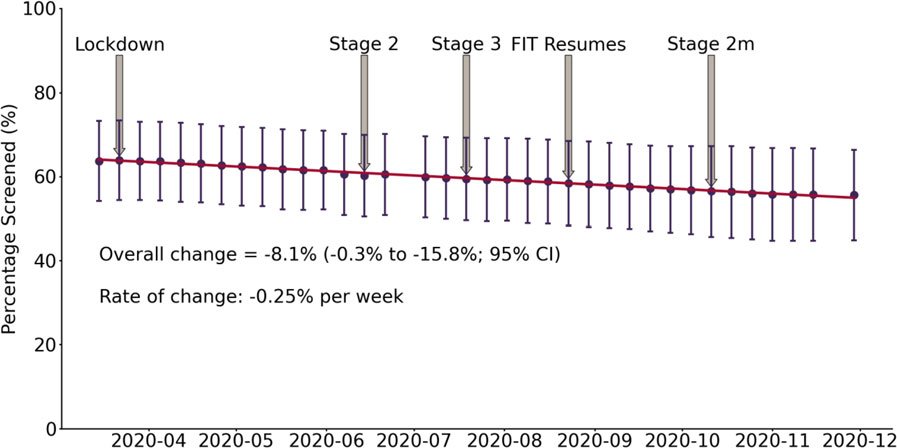
Colorectal Cancer Screening Rates. Shows the mean percentage (percentage screened ± SD) of eligible patients up to date for colorectal cancer screening each week (*n* = 7170 March 15, 2020; *n* = 7168 November 29, 2020). For reference the lockdown and re-opening stages and when FIT testing could be ordered again have been identified. Lockdown = March 20, 2020, Stage 2 = June 12, 2020, Stage 3 = July 17, 2020, FIT Resumes = August 25, 2020, and Modified Stage 2 = October 13, 2020

**Fig. 3:**
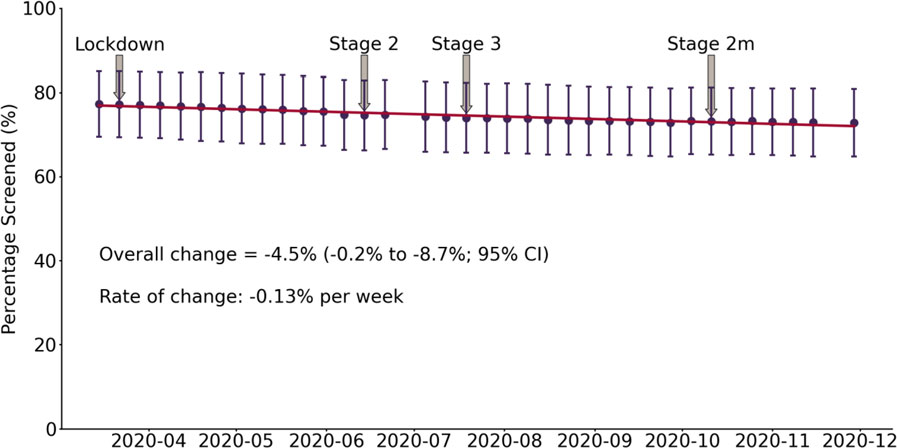
Type 2 Diabetes Screening Rates. Shows the mean percentage (percentage screened ± SD) of eligible patients up to date for cervical cancer screening each week (*n* = 10,897 on March 15, 2020; n = 10,933 on November 29, 2020). For reference the lockdown and re-opening stages have been identified. Lockdown = March 20, 2020, Stage 2 = June 12, 2020, Stage 3 = July 17, 2020, and Modified Stage 2 = October 13, 2020

During the 38 weeks since March 15, 2020, the mean preventive care screening rates decreased for cervical cancer screening (Fig. 1), colorectal cancer screening (Fig. 2), and type 2 diabetes screening (Fig. 3). Cervical cancer screening rates decreased by 7.5% (− 0.3% to − 14.7%; 95% CI). The mean colorectal cancer screening rates decreased by 8.1% (− 0.3% to − 15.8%; 95% CI). The mean type 2 diabetes screening rates decreased by 4.5% (− 0.2% to − 8.7%; 95% CI).

A line of best fit was generated for each screening test and the slope indicated the weekly rate of change during this period. Cervical cancer screening rates decreased by 0.23% per week (Fig. 1). Colorectal cancer screening rates decreased by 0.25% per week (Fig. 2). Type 2 diabetes screening rates decreased by 0.13% per week (Fig. 3).

The Government of Ontario’s stages of gradual reopening are indicated for reference to demonstrate the impact of re-opening efforts on screening rates (Figs. 1, 2, and 3). For colorectal cancer screening, the date that FIT testing could be ordered was also shown (Fig. 2). None of the re-opening measures reversed the decreased screening trends across the three observed screening tests.

### Crude estimates of the preventive care service deficits from baseline screening

The number of patients among the 22,685 active patients at the three clinics requiring screening to return to baseline, low-risk screening rates were determined. A total of 505 (20 to 993; 95% CI) patients would need to have Papanicolaou smear testing (Table 4), 577 (22 to 1133; 95% CI) would need CRC screening (Table 5), and 489 (21 to 951; 95% CI) would need type 2 diabetes screening (Table 6).

**Table 4:** Crude Estimate Number of Patients Due for Cervical Cancer Screening

	No. in Age Range	% Eligible for Screening	# Eligible for Screening	% Due for Screening (Baseline)	No. Due for Screening	LCL 95% of % Due for Screening	LCL No. Due for Screening	UCL 95% of % Due for Screening	UCL No. Due for Screening
Study Clinics	7246	93.2%	6754	7.5%	505	0.3%	19	14.7%	990
Ontario	4,130,515	93.2%	3,850,000	7.5%	288,000	0.3%	11,000	14.7%	565,000
Canada	10,734,670	93.2%	10,006,000	7.5%	748,000	0.3%	28,000	14.7%	1,467,000

The number of females in the screening age range for cervical cancer screening were calculated from 2016 census data [20]. The percentage of patients eligible for screening and due for screening to return to baseline were determined from the study clinics, then used to estimate the number of patients eligible for screening in Ontario and Canada. The lower confidence level (LCL) and upper confidence level (UCL) were also determined based on the study population. Estimations for Ontario and Canada were rounded to the nearest 1000 patients

**Table 5:** Crude Estimate Number of Patients Due for Colorectal Cancer Screening

	No. in Age Range	% Eligible for Screening	# Eligible for Screening	% Due for Screening (Baseline)	No. Due for Screening	LCL 95% of % Due for Screening	LCL No. Due for Screening	UCL 95% of % Due for Screening	UCL No. Due for Screening
Study Clinics	7368	97.3%	7168	8.1%	577	0.3%	24	15.8%	1130
Ontario	4,158,340	97.3%	4,045,000	8.1%	326,000	0.3%	13,000	15.8%	638,000
Canada	10,982,180	97.3%	10,006,000	8.1%	860,000	0.3%	35,000	15.8%	1,685,000

The number of patients in the screening age range for colorectal cancer screening were calculated from 2016 census data [20]. The percentage of patients eligible for screening and due for screening to return to baseline were determined from the study clinics, then used to estimate the number of patients eligible for screening in Ontario and Canada. The lower confidence level (LCL) and upper confidence level (UCL) were also determined based on the study population. Estimations for Ontario and Canada were rounded to the nearest 1000 patients

**Table 6:** Crude Estimate Number of Patients Due for Type 2 Diabetes Screening

	No. in Age Range	% Eligible for Screening	# Eligible for Screening	% Due for Screening (Baseline)	No. Due for Screening	LCL 95% of % Due for Screening	LCL No. Due for Screening	UCL 95% of % Due for Screening	UCL No. Due for Screening
Study Clinics	12,402	88.2%	10,933	4.5%	489	0.2%	23	8.7%	954
Ontario	6,952,870	88.2%	6,129,000	4.5%	274,000	0.2%	13,000	8.7%	535,000
Canada	18,139,570	88.2%	15,991,000	4.5%	714,000	0.2%	34,000	8.7%	1,396,000

The number of patients in the screening age range for type 2 diabetes were calculated from 2016 census data [20]. The percentage of patients eligible for screening and due for screening to return to baseline were determined from the study clinics, then used to estimate the number of patients eligible for screening in Ontario and Canada. The lower confidence level (LCL) and upper confidence level (UCL) were also determined based on the study population. Estimations for Ontario and Canada were rounded to the nearest 1000 patients

Extrapolation of these numbers to Ontario’s population provided a crude estimate of the Ontarians requiring screening to return to pre-COVID-19, low-risk screening rates. Potentially 288,000 Ontarians (11,000 to 565,000; 95% CI) would need Papanicolaou smear testing (Table 4), 326,000 Ontarians (13,000 to 638,000; 95% CI) would need CRC screening (Table 5), and 274,000 Ontarians (13,000 to 535,000; 95% CI) would need T2DM screening (Table 6).

Similarly, extrapolation to the Canadian population provided a crude estimate of Canadians needing screening to return to pre-COVID-19, low-risk screening rates. Potentially 745,000 Canadians (28,000 to 1,467,000; 95% CI) would need Papanicolaou smear testing (Table 4), 860,000 Canadians (35,000 to 1,685,000; 95% CI) would need CRC screening (Table 5), and 715,000 Canadians (34,000 to 1,396,000; 95% CI) would need T2DM screening (Table 6).

### Interpretation

This observational cohort study demonstrates that preventive care service delivery was negatively affected by COVID-19 at the study clinics. This trend has not reversed with re-opening measures implemented by the Government of Ontario. This finding is supported by an abrupt decrease in screening volumes across Ontario for CRC and cervical cancer peaking at 91.3 and 92.3% reductions, respectively [2]. These decreased screening volumes were still below baseline levels as of December 2020 [2], which aligns with persistent decrease in screening demonstrated in this study. Here we provide crude estimates, which indicate that hundreds of thousands of Ontarians and Canadians may have delayed or been unable to access preventive care services since March 2020.

According to a review of preventive care services, only cervical cancer screening requires in-person assessments [5]. Therefore, the finding that cervical cancer screening decreased since March 2020 is as expected, because Ministry of Health directives recommended avoiding in-person assessments [21]. However, CRC screening may be offered through virtual care [5]. The observed decrease in CRC screening is not explained by fewer in-person appointments. Instead, both laboratories pausing FIT kit distribution to reserve capacity for COVID-19 testing [10] and the Ministry of Health recommendation to defer non-essential services [21] have likely contributed to decreased CRC screening. This recommendation to defer healthcare services has likely reduced T2DM screening, as screening could be offered through virtual care [5] without restrictions on hemoglobin A1c or fasting blood sugar testing. Similar findings of reduced utilization of healthcare services have been observed through fewer emergency department visits for heart failure [22], stroke [23], and pediatric assessments [24]. Patient and provider clinical priorities may also shift from prevention and screening to management of active problems, like increased demand for mental healthcare services [25]. Therefore, the findings of this study indicate that the reductions in preventive care service delivery are likely multifactorial.

Sustained reductions in preventive care are concerning since screening can detect early disease like cervical cancer, CRC, or T2DM. Accordingly, many cases of early disease are likely going undetected. Delayed diagnoses may have significant consequences as each 4 week delay in CRC treatment could increase mortality rates by 6 to 8% [3]. This is supported by a recent model that predicted prolonged preventive care delays will cause higher cancer mortality and advanced disease at diagnosis [26]. From a health system perspective, delayed cancer diagnoses may significantly increase cancer treatment costs [27]. Prolonged undiagnosed and untreated T2DM is also expected to present problems since untreated T2DM increases the risk of cardiovascular disease mortality [28]. Therefore, strategies to restore preventive care service delivery to pre-COVID-19 levels are essential. As COVID-19 restrictions persist and recur, the multifactorial patient, provider and health system factors impacting preventive care delivery need to be better understood and addressed.

This study has demonstrated that EMR data can be used to determine patients’ preventive care screening statuses. This automated function could be developed for other EMRs to generate monthly preventive care reports for providers [29]. These reports could then be used for targeted preventive care delivery, prioritising in-person visits for those most overdue or needing tests that require in-person assessments. Additionally, point-of-care tools could support opportunistic preventive care delivery during visits for other reasons. The literature supports that digital solutions like EMR reminders [30] combined with active panel management [29, 30] can improve screening rates. However, in order to leverage EMR data to improve preventive care rates, system capacity must be improved as laboratories have only recently restored some capacity for screening test [31].

Another potential strategy has already been implemented by the Government of Ontario to address the surgical backlog, including cancer surgeries [32]. This strategy involves investments in system capacity and providing financial incentives for health service delivery [33]. Directing additional resources upstream to support additional time and planning to restore preventive care services could effectively boost screening rates [34] and maintain early disease detection. This could help mitigate the anticipated increase in cancer mortality, later stage diagnoses [26], and increased health care costs [27]. These resources may also maintain the focus on early detection as large numbers of patients begin seeking care for neglected physical and mental conditions [35].

### Limitations

This study extrapolated data based on three clinics in Ottawa, a single urban centre in Ontario, Canada. The data in Table 2 indicates that the study clinic age demographics do not exactly match the Ontario and Canadian populations. As well, differences may exist between urban and rural preventive care service delivery and across Ontario. Lastly, each province is responsible for the local health care service delivery. Therefore, the generalizability could be impaired, and attempts have been made to avoid overstating the results beyond the local clinic. This study also relies on high quality EMR data which may suffer from accuracy, completeness, and consistency issues [36]. These challenges arose when developing the data export. Screening data was not consistently encoded following the same method, therefore multiple exported data points had to be amalgamated to improve data quality. Lastly, the impact of COVID-19 on T2DM screening may be underestimated since a 3 year interval was used despite guidelines recommended 1 to 3 years based on calculated risk scores.

## Conclusions

This observational cohort study estimated that hundreds of thousands of Canadians may not have been screened for cervical cancer, CRC, and T2DM according to low-risk screening guidelines. Re-opening initiatives have not reversed the decrease in screening rates. Given the decreased screening, Canadians will likely be facing a surge of later stage cancer and diabetes diagnoses. Therefore, strategies like using EMR data to inform active panel management and directing additional resources to preventive care delivery and testing will be needed to reverse these trends and catchup on the hundreds of thousands of overdue tests.

## Supplementary Information

**Additional file 1.**

**Additional file 2.**

**Additional file 3.**

**Additional file 4.**

## Data Availability

The datasets generated and/or analysed during the current study are not publicly available due patient confidentiality and privacy. Filtered data may be available from the corresponding author on reasonable request and review by Bruyère Research Ethics Board.
