# Supplementary material for: Estimated impact of COVID-19 on preventive care service delivery: an observational cohort study
Source: BMC Health Serv Res. 2021 Oct 16;21:1107. doi: 10.1186/s12913-021-07131-7 (PMC8520349; doi:10.1186/s12913-021-07131-7)
Supplement: Supplementary file 1 — Additional file 1. [file 12913_2021_7131_MOESM1_ESM.docx]

**Appendix 1. Cervical Cancer Inclusion and Exclusion Criteria**

Inclusion Criteria (Low-Risk)

1. Age ≥ 25 years and Age ≤ 69 years
2. Female sex

Exclusion Criteria (High-Risk or Ineligible for Screening)

1. Past medical history documents total hysterectomy using:
   1. “hysterectomy”, “tah”, “tahbso”, “tvh”, “tabh”
   2. But is not a partial hysterectomy:
      1. “partial hysterect”
2. Past medical history documents cervical cancer using:
   1. “cervical ca”, “cerv ca”
   2. But is not documentation of cervical cancer screening using:
      1. “cervical ca screen”, “cervical cancer screen”, “screening for cerv”, or “screening cerv”
3. Past medical history indicates that a pap test is not indicated
   1. “pap not medically indicated”
4. Patient’s data marked as “private”
