# Supplementary material for: Estimated impact of COVID-19 on preventive care service delivery: an observational cohort study
Source: BMC Health Serv Res. 2021 Oct 16;21:1107. doi: 10.1186/s12913-021-07131-7 (PMC8520349; doi:10.1186/s12913-021-07131-7)
Supplement: Supplementary file 2 — Additional file 2. [file 12913_2021_7131_MOESM2_ESM.docx]

**Appendix 2. Colorectal Cancer Inclusion and Exclusion Criteria**

Inclusion Criteria (Low-Risk)

1. Age ≥ 50 years and Age ≤ 74 years

Exclusion Criteria (High-Risk or Ineligible for Screening)

1. Past medical history documents previous colorectal cancer:
   1. 'colon ca', 'rectal ca', 'colon adeno', 'rectal adeno'
   2. But is not documentation of colorectal cancer screening using:
      1. 'colon ca screen', 'colon cancer screen', 'screening for colo', 'screening colorec', 'colon cancer check'
2. Past medical history documents diagnosis of inflammatory bowel disease using:
   1. 'ulcerative proctitis', 'ulcerative colitis', 'crohn', 'chron's', 'ibd', 'inflammatory bowel'
3. Past medical history documents the patient has had a colectomy using:
   1. 'colectomy'
4. Past medical history documents a positive family history of colorectal cancer:
   1. 'family history colon ca', 'family hx colon ca', 'fam hx colon',
5. Patient’s data marked as “private”
