# Supplementary material for: Estimated impact of COVID-19 on preventive care service delivery: an observational cohort study
Source: BMC Health Serv Res. 2021 Oct 16;21:1107. doi: 10.1186/s12913-021-07131-7 (PMC8520349; doi:10.1186/s12913-021-07131-7)
Supplement: Supplementary file 3 — Additional file 3. [file 12913_2021_7131_MOESM3_ESM.docx]

**Appendix 3. Type 2 Diabetes Inclusion and Exclusion Criteria**

Inclusion Criteria (Minimum Screening Interval)

1. Age ≥ 40 years

Exclusion Criteria (High-Risk or Ineligible for Screening)

1. Past medical history documents a diagnosis of type 2 diabetes:
   1. 'diabetes', 't2dm', 'diabetic', 'type 2 dia', 'type ii dia', 'dm2', 'dmii'
   2. But patient is not pre-diabetic or had gestational diabetes:
      1. 'prediabet', 'pre-diabet', 'gestational diabet'
2. Latest Hemoglobin A1c result ≥ 6.5%
3. Latest Fasting Blood Glucose result ≥ 7 mmol/L
4. Patient’s data marked as “private”
