# Supplementary material for: Estimated impact of COVID-19 on preventive care service delivery: an observational cohort study
Source: BMC Health Serv Res. 2021 Oct 16;21:1107. doi: 10.1186/s12913-021-07131-7 (PMC8520349; doi:10.1186/s12913-021-07131-7)
Supplement: Supplementary file 4 — Additional file 4. [file 12913_2021_7131_MOESM4_ESM.docx]

**Appendix 4.** Preventive Care Data Points Extracted from Each Patient Record.

| Screening Type | Test Type | Data Points |
| --- | --- | --- |
| Cervical Cancer | Papanicolaou Smear | - pat.Diagnostic_Test_Reports.Pap_Test_Report.months_since_latest - pat.Lab_Values.Pap_Smear_[Pap_Smear].months_since_latest - pat.Lab_Text.Lab_Text_Containing"Note:_pap_tests_help_screen_for_cervical_cancer_and_its_precursors;".months_since_latest - pat.Lab_Text.Lab_Text_Containing"papanicolaou_smear_liquid_based".date_of_latest |
| Colorectal Cancer (CRC) | Fecal occult blood test (FOBT) | - pat.Lab_Values.Stool_Occult_Blood_[Stool_Occult_Blood].months_since_latest |
|  | Fecal immunochemical test (FIT) | - pat.Lab_Values.Fecal_Immunochemical_Test_[FIT].months_since_latest |
|  | Sigmoidoscopy | - pat.Diagnostic_Test_Reports.Sigmoidoscopy.months_since_latest |
|  | Colonoscopy | - pat.Diagnostic_Test_Reports.Colonoscopy.months_since_latest |
| Type 2 Diabetes Mellitus (T2DM) | Hemoglobin A1c (HbA1c) Date | - pat.Lab_Values.Hemoglobin_A1C_[Hb_A1C].months_since_latest |
|  | HbA1c Result | - pat.Lab_Values.Hemoglobin_A1C_[Hb_A1C].latest_value |
|  | Fasting Blood Sugar (FBS) Date | - pat.Lab_Values.Fasting_Blood_Sugar_[FBS].months_since_latest |
|  | FBS Result | - pat.Lab_Values.Fasting_Blood_Sugar_[FBS].latest_value |
